# Tissue Specificity in Social Context-Dependent *lysozyme* Expression in Bumblebees

**DOI:** 10.3390/antibiotics9030130

**Published:** 2020-03-20

**Authors:** H. Michael G. Lattorff

**Affiliations:** 1 International Centre of Insect Physiology and Ecology (icipe), Nairobi 00100, Kenya; mlattorff@icipe.org; Tel.: +254-20-863-2066; 2 Naturwissenschaftliche Fakultät I, Martin-Luther-Universität Halle-Wittenberg, 06099 Halle (Saale), Germany; 3 German Centre for Integrative Biodiversity Research (iDiv) Halle-Jena-Leipzig, 04103 Leipzig, Germany

**Keywords:** cage experiment, social environment, social context, *Bombus terrestris*, social immunity

## Abstract

Group living at high densities may result in the enhanced transmission of pathogens. Social insects are obligate group-living species, which often also exhibit high relatedness and frequent social interactions amongst individuals, resulting in a high risk of disease spread. Social species seem to exhibit immune systems that provide colonies of social insects with a certain level of flexibility for adjustment of immune activity according to the risk of disease spread. In bumblebees, *Bombus terrestris*, it was demonstrated that in group-kept individuals, immune component activity and immune gene expression is increased, potentially as a prophylactic adaptation. Here, I tested whether social environment influences the gene expression pattern of two *lysozyme* genes, which are components of the antimicrobial response of the bumblebee. In addition, I tested gene expression activation in different tissues (gut, fat body). The analysis revealed that the gene, the density of individuals, the tissue, and the interaction of the latter are the main factors that influence the expression of *lysozyme* genes. This is the first report of a tissue-specific response towards the social environment. This has implications for gene regulation, which must be responsive to social context-dependent information.

## 1. Introduction

Social insects typically live in colonies in which individuals are highly related and show frequent social interactions [1,2]. This provides excellent opportunities for parasites and pathogens to spread and establish themselves [3]. Besides their individual immune system which consists of the canonical humoral immune pathways and cellular immune responses, social insects show a range of adaptations at the group level known as social immunity [4]. These consist of behavioural and physiological adaptations to combat pathogens and parasites.

The humoral immune systems of social insects have been shown to be impaired by a loss of genes [5]. However, recent evidence shows that the loss of genes might precede the evolution of sociality since a solitary bee (*Megachile rotundata*) shows a similar lack of immune genes as the social species [6]. Several hypotheses have been put forward that aim at explaining how social insects might compensate for the lack of immune genes. Social immunity, neofunctionalization of genes not associated with immunity in other insects, but also changes in gene regulation might compensate for the lack of immune genes.

Group level effects on the activity of immune system components [7] and immune gene expression have been shown in several systems [8,9]. Effector genes of the innate immune system might be expressed at higher levels in larger groups, potentially as the risk of infection increases [8]. Antimicrobial effector genes are expressed in the fat body of the insect and released into the hemolymph. The gut is another tissue with high levels of antimicrobial effector gene expression, in particular midgut epithelial cells [10,11].

The bumblebee (Bombus sp.) is well-suited for experiments related to the analysis of group-level effects on immune function. Their colonies are headed by a single, once-mated queen so that all individuals are super-sisters and hence, due to the haplo-diploid system of Hymenoptera, are related by r = 0.75 to each other. Colonies are annual and easy to manipulate in the lab.

Here, I test whether different *lysozyme* genes expressed in different tissues are regulated depending on the social context. In previous experiments, *lysozyme* genes showed a strong response towards the social context by strong down-regulation of expression in individuals kept solitary [8]. However, previous experiments used the whole abdomen, which did not allow for the separation of the effects of fat body derived gene expression and gut derived gene expression. Here, I manipulated the group size and the density of bees in hoarding cages kept under controlled laboratory conditions and subsequently tested separately for the expression of different *lysozyme* genes in the gut and fat body tissue.

## 2. Results

**Figure 1:**
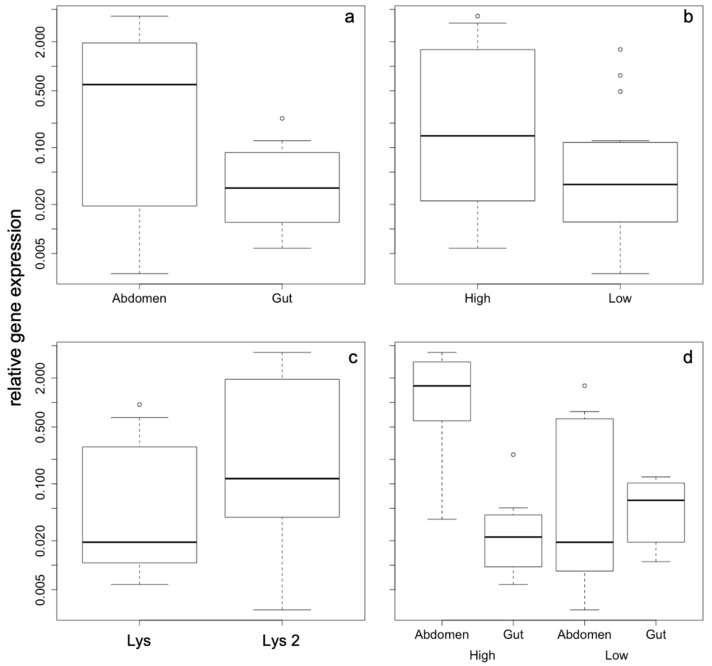
Relative gene expression as a function of the factors that were included in the best model with the following factors: (**a**) tissue, (**b**) density, (**c**) gene, and (**d**) tissue x density interaction. A TukeyHSD test revealed that gut/high vs. abd/high, abd/high vs. abd/low, and gut/low vs. abd/high are significantly different at *p* < 0.05, while all other comparisons are not significant. All axes are displayed in logarithmic scale.

The relative gene expression levels of the two *lysozyme* genes showed variable expression influenced by density, gene, tissue, and density × tissue interaction. These factors were included in the best model of the model selection procedure. In Table 1, the results of the model averaging are displayed with the factors contained in the final, all being highly significant (tissue *p* = 4 × 10^−5^; density/tissue *p* = 0.0006; density *p* = 0.004) with the exception of the factor gene, which was marginally significant (*p* = 0.06). The overall differences in relative gene expression according to the factors contained in the best model are shown in Figure 1a–d.

**Figure 2:**
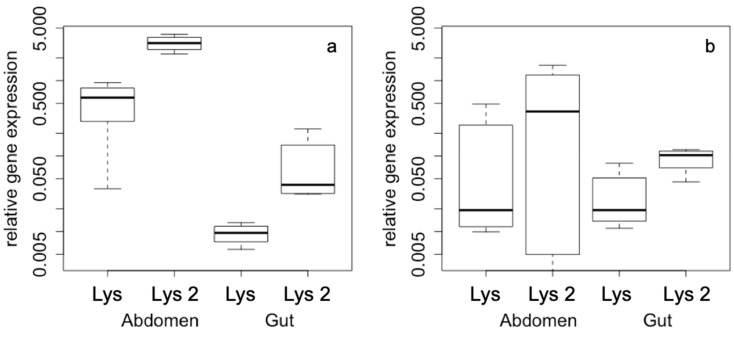
Relative gene expression according to (**a**) high density and (**b**) low density conditions with gene (*Lys* and *Lys-2*) and tissue (abdomen, gut) as factors. All axes are displayed in logarithmic scale.

The most pronounced effects of gene expression differences were found according to the density of the animals within the cage irrespective of the group size. Density also results in tissue-dependent expression pattern, whose differences are more strongly expressed under high-density conditions. Abdominal expression of both *lysozyme* genes is highly up-regulated compared to gut expression (Figure 2a), while under low-density conditions there are no differences in gene expression pattern, neither for the two genes nor for the different tissues (Figure 2b).

## 3. Discussion

Gene expression responses of antimicrobial active genes resulting in the production of antimicrobial peptides are characterized by flexibility, which is known, amongst others, as tissue specificity [12]. Here, I demonstrate that immune gene expression is not only characterized by tissue specificity, but also by social context. *Lysozymes* are enzymes that hydrolyse the glycosidic linkage between *N*-acetylmuramic acid and *N*-acetylglucosamine in peptidoglycan, a part of the cell wall of bacterial cells. These enzymes therefore cause the lysis of the bacterial cell [13]. They are regulated according to social context, which was manipulated in terms of the density of individuals and according to tissue, as well as their interaction. *Lysozymes* were up-regulated under high-density conditions and in the abdomen (fat body tissue).

In insects that build temporary or even obligate aggregations, an increased risk of disease transmission is expected to occur. Hence, prophylactic responses of the innate immune system are expected to deal with this increased risk of infection under high-density conditions. In social bees, it has been shown experimentally that social context indeed influences flexible immune activation responses. Phenol oxidase activity and antibacterial activity of the hemolymph is increased in individuals kept in groups compared to those kept solitary [7]. Furthermore, it was shown that genes responsible for both the phenoloxidase system as well as for antibacterial responses are regulated at the level of gene expression, with up-regulated gene expression in group-kept individuals compared to those kept solitary [8].

As a line of defence, insects have several barriers against intruding pathogens. As many pathogens might be taken up with the food, the gut, especially the midgut epithelium, builds a line of defence in the form of a physical barrier. Several lines of chemical defence are also present in the midgut, e.g., the antioxidant defence (reactive oxygen species are attacked) and nitric oxide systems, but the expression of antimicrobial active substances, e.g., anti-microbial peptides (AMPs) and *lysozymes*, is also known as a defensive system.

Antimicrobial active substances are also expressed within the fat body, a tissue located within the abdomen of insects, analogous to the vertebrate liver. The antimicrobial substances are released into the hemolymph through which they are distributed within the insect body. Overall, an abdominal increase in antimicrobial activity [7] and the expression of antimicrobial genes [8] might be a result of either gut or fat body activity, or even both of them combined. As previous studies have not addressed these issues, I will now show that in bumblebees the abdominal tissue (fat body) is responsive to the social context, while the gut tissue does not show any social context-related changes to gene expression.

The social context might be difficult to define, as it might relate to group size or to differences in the density of individuals. Here, I used experimental variation in both, and the results indicated that the density of individuals is particularly important, as variation in group size did not have significant effects on the regulation of antimicrobial gene expression. This finding is in agreement with another study that analysed stress responses in caged honeybee workers. Here, group size effects were not detectable [14].

Insects, especially group-living insects, have adapted to the increased risk of disease transmission under high-density conditions by increased activity of immune system components. Different social bee species, as well as thrips species varying in the degree of social complexity and of group size, show different levels of antimicrobial activity of their hemolymph [15,16]. Social species show additional adaptations. First, they show higher rates of molecular evolution of immune genes [17,18,19,20]. However, social species not only possess a hard-wired increased activity but also a flexible way of increasing immune responses in a prophylactic way dependent on the local social context [7,8]. At least for *lysozyme* genes, there is a tissue specificity in the prophylactic immune system activation where abdominal (fat body) expression is responsive to the social context, while gut expression is not responsive to social context.

This has two further implications. Firstly, there might be specific gene expression activation targets (promotor regions) that are responsive to the social context. Secondly, it is necessary to understand how the perception of the social context, which is mediated by the peripheral nervous system through visual or chemical stimuli, is transmitted to other non-nervous system derived organs in the periphery of the organism.

## 4. Materials and Methods

### 4.1. Bee Samples and Experimental Manipulation

Bumblebee (Bombus terrestris) workers were obtained from a colony of a commercial supplier (Koppert BV, Berkel en Rodenrijs, The Netherlands). The colony was supplied with sugar syrup (30%, *w*/*v*) ad libitum and kept inside a dark room at 28 °C and 60% relative humidity. Colony inspections and sampling of workers were done under red light conditions to reduce disturbance of the bees. Workers were sampled at random and transferred into hoarding cages (9.5 × 8.5 × 5.5 cm and 14.5 × 12 × 5 cm). Cages were supplied with sugar candy and pollen as a protein source. In addition, cages were supplied with water. I inspected the cages daily to exchange food and water. Dead bees were removed and replaced by new bees kept under similar conditions. Cages were kept under similar environmental conditions to the ones described for the colonies.

For the treatments that aimed at varying the social context, I manipulated the size and density of the groups kept in the cages. Small (9.5 × 8.5 × 5.5 cm) and large cages (14.5 × 12 × 5 cm) supplemented with either five or ten workers were used. Bees were kept for 10 days under these experimental conditions and at the end of the time period workers were immediately transferred to liquid nitrogen and subsequently stored at −80 °C until further processing. I used four replicates for each of the experimental conditions (group size, density) and from each cage one individual was chosen randomly for subsequent gene expression analysis (*n* = 16).

### 4.2. RNA Extraction and Gene Expression Analysis

Individual bee samples were dissected under a stereomicroscope. While the bees were still frozen, their abdomens were cut open using a scalpel in order to remove the gut. The gut was immediately transferred into 500 µL Trizol (Qiagen, Hilden, Germany). The remaining abdomens were separated from the rest of the bodies and transferred into 500 µL Trizol (Qiagen, Germany). Subsequently, total RNA was extracted according to a standard protocol [21] with slight modifications [8,22]. Finally, extracted RNA was dissolved in 20 µL DEPC-water (0.1% *v/v* DEPC). The quality and quantity of the RNA were determined using a spectrophotometer (Nanodrop 1000, peqlab, Erlangen, Germany). cDNA synthesis was done using 1 µg total RNA, 80 U M-MLV Reverse Transcriptase (Promega, Mannheim, Germany) and 0.8 µg Oligo (dT)_15_ Primer (Promega, Mannheim, Germany). SureClean Plus (Bioline, Luckenwalde, Germany) was used to purify cDNA and subsequently re-dissolved in 20 µL DEPC-water (0.1% *v/v* DEPC). Nanodrop 1000 (peqlab, Erlangen, Germany) was used to determine the quality and quantity of cDNA samples.

Quantitative RT-PCR was used for the quantification of the gene expression of the target genes. Two different target genes were used here, *lysozyme* (LOC100642297) and *lysozyme 2* (LOC100649284). cDNA (concentration: 10 ng/µL) was used in a reaction containing: 1 µL cDNA, 5 µL SensiMixPlus SYBR & Fluorescein Kit (Bioline, Luckenwalde, Germany), 0.3 mM of each gene-specific primer and 3.4 µL DEPC-H_2_O (0.1% ***v/v*** DEPC). Primers were adopted from previous studies: *Lys* (fwd: 5′-ACGCAGTGTGAAGCCGTGCAGGA-3′; rev: 5′-AGCTGGAGGCAGTCTTCGGACCAGT-3′) and *Lys-2* (fwd: 5′-GGATCTGTTTGTGGACCATT-3′; rev: 5′-TAATGCCATCACCGTTACAG-3′) from [8] and the housekeeping gene 28S rRNA (fwd: 5′-TCGGTCTACGGCCCGAGTGG-3′; rev: 5′-GCGGTCCAGACGCACACACA-3′) is from [22]. The thermal profile used was chosen as follows: 1 cycle at 95 °C for 10 min, followed by 39 cycles at 95 °C for 15 s (denaturation), 57 °C for 30 s (primer annealing) and 72 °C for 30 s (elongation) using a CFX ConnectTM Real-Time PCR Detection System (Bio-Rad, Munich, Germany). All samples were amplified in duplicates. Melting curve analysis was carried out to verify the specificity of PCR amplification by reading fluorescence at 1 °C intervals for the temperature range between 55 °C and 98 °C. All PCRs showed only a single peak in the melting curve analysis, indicative of the high specificity of the primers of all target and housekeeping genes. All samples exhibiting differences between replicates of 0.5 C_t_-values or higher were replicated twice using the same cDNA sample.

### 4.3. Data Analysis

Gene expression quantification was carried out by means of relative expression utilizing a house-keeping gene (HKG) to standardize the gene expression. HKGs are expected to be unaffected by treatment (social context) or tissue (gut, abdomen). Here, I used the gene for 28S rRNA, which is a suitable HKG in the bumblebee *Bombus terrestris* [8].

Relative gene expression was determined by incorporating the PCR efficiency for every gene calculated by LinReg [23]. The ΔCt method [24] was used with slight modifications accounting for PCR efficiency [22].

Relative gene expression levels for the two *lysozyme* genes were log-transformed as untransformed data deviated from a normal distribution. Using the log-transformed relative gene expression levels as a response variable, I tested for the influence of several factors (gene, tissue, group size, density and all possible interaction terms) using an ANOVA with model selection (*dredge* function) and model averaging (*model.avg* function) using the R package *MuMIn* [25]. A TukeyHSD post hoc test was used to infer significance of within-factor combinations. All data analyses, including the statistical analyses, were carried out using standard spreadsheet software and R v3.5.3 [26].

## Figures and Tables

**Table 1:** Results of model averaging. The factors included in the best model after model selection are shown in bold letters.

Factor	Estimate	S.E.	Adj. S.E.	z Value	Pr (>|z|)
(intercept)	0	0	0	NA	NA
**Density**	−0.696	0.233	0.244	2.850	0.0044
**Gene**	0.440	0.227	0.237	1.858	0.0632
**Tissue**	−0.905	0.210	0.221	4.093	4.2 × 10^−5^
**Density × Tissue**	0.755	0.211	0.221	3.412	0.0006
**Group size**	0.162	0.165	0.173	0.937	0.3488
**Density ×** Gene	−0.214	0.210	0.221	0.969	0.3327
**Gene ×** Group size	−0.231	0.392	0.412	0.560	0.5752
**Gene ×** Tissue	0.025	0.212	0.223	0.110	0.9124
**Density ×** Group size	0.076	0.438	0.460	0.166	0.8685
**Group size ×** Tissue	0.068	0.401	0.422	0.162	0.8716
